# Serum lipid biomarkers and inflammatory cytokines associated with onset and clinical status of patients with early knee osteoarthritis

**DOI:** 10.3389/fnut.2023.1126796

**Published:** 2023-03-15

**Authors:** Luz Herrero-Manley, Ana Alabajos-Cea, Luis Suso-Martí, Ferran Cuenca-Martínez, Joaquín Calatayud, José Casaña, Enrique Viosca-Herrero, Isabel Vázquez-Arce, Francisco José Ferrer-Sargues, María Blanco-Díaz

**Affiliations:** ^1^Servicio de Medicina Física y Rehabilitación, Hospital La Fe, Valencia, Spain; ^2^Grupo de Investigación en Medicina Física y Rehabilitación, Instituto de Investigación Sanitaria La Fe (IISLAFE), Valencia, Spain; ^3^Exercise Intervention for Health Research Group (EXINH-RG), Department of Physiotherapy, University of Valencia, Valencia, Spain; ^4^Department of Physiotherapy, University of Valencia, Valencia, Spain; ^5^Department of Physiotherapy, Universidad Cardenal Herrera-CEU, CEU Universities, Valencia, Spain; ^6^Department of Surgery and Medical Surgical Specialties, Faculty of Medicine and Health Sciences, University of Oviedo, Oviedo, Spain

**Keywords:** osteoarthritis, early osteoarthritis, serum lipid, inflammatory biomarkers, cytokine

## Abstract

**Introduction:**

Osteoarthritis (OA) is a common joint condition and one of the greatest causes of disability worldwide. The role of serum lipid and inflammatory biomarkers in the origin and development of the disease is not clear, although it could have important implications for diagnosis and treatment. The primary aim of this study was to evaluate differences of serum lipid and inflammatory biomarkers with knee EOA in comparison with matched controls, in order to determine the role of these factors in the origin of EOA.

**Methods:**

For this proposal, a cross-sectional study with a non-randomized sample was performed. 48 subjects with early osteoarthritis (EOA) and 48 matched controls were selected and serum lipid levels (total cholesterol, LDL, HDL) and inflammatory biomarkers C-reactive protein (CRP), uric acid (UA) were analyzed. In addition, clinical (pain, disability) and functional (gait speed, sit-to-stand) variables were measured to establish their relationship to serum lipid levels and inflammatory biomarkers.

**Results:**

Patients with EOA showed higher levels of total cholesterol LDL, UA, and CRP. Higher levels of total cholesterol, LDL and CRP were correlated with higher levels of pain intensity and higher disability (*p* < 0.05). In addition, UA and CRP were inversely correlated with gait speed and sit-to-stand tests (*r* = −0.038 to −0.5, *p* < 0.05).

**Conclusion:**

These results highlight the relevance of metabolic and proinflammatory aspects in the early stages of knee OA and could be key to developing early diagnoses to prevent the onset and development of the disease.

## Introduction

Osteoarthritis (OA), one of the 10 main causes of physical disability today, has become a major public health problem worldwide, with knee osteoarthritis being the predominant form of this injury (1). Clinical presentation of knee OA includes degenerative changes due to the progressive loss of articular cartilage, subchondral bone changes, synovial inflammation, and meniscus degeneration (2, 3). All these manifestations lead to joint and multisite pain, stiffness, and loss of range of movement, affecting physical function and quality of life of patients (4).

A directly proportional relation between knee OA prevalence and demographic factors such as gender, aging and obesity rates has been found (5). However, these parameters, evaluated as isolated risk factors, are not the specific cause of OA in most cases (6). Currently, a new paradigm in OA has been proposed, shifting from a cartilage-specific disease toward that of an organ disease, in which synovitis and systemic pathology plays an important role (7, 8). Clustering of cardiovascular and metabolic factors can increase the risk of OA, causing a condition identified as metabolic syndrome (9, 10). This syndrome includes alterations of blood vessel function, decreased high-density lipoprotein (HDL) levels, increased low-density lipoprotein (LDL) and triglyceride levels, raised blood glucose levels and abdominal obesity, creating an archetype of low-grade chronic inflammation (11). The systemic nature of OA allows for potential future treatments that could improve the development of the disease and the quality of life of people suffering from the disease (12, 13).

Several theories have tried to explain the pathogenesis of OA and its relationship with the different components of metabolic syndrome. Despite underlying differences between them, a consensus has been reached that mechanical factors and humoral mediators are involved in the course of the disease (14, 15). The metabolic imbalance characteristic of metabolic syndrome, produced by the accumulation of adipose tissue, increased vascular resistance and insulin resistance, among other factors, produces alterations in protein concentrations, ultimately triggering these inflammatory processes (16).

Multifactorial causes of OA, and the combination of mechanical and metabolic variables, determine that the structural findings of the disease are a late phenomenon (17, 18) This process has a negative impact on patients, since non-surgical interventions normally have limited efficacy, since they are applied at an advanced stage of the pathology with severe joint and functional involvement (19, 20) In addition, the lack of control over the triggering factors translates into inaccurate forecasts, loss of functional independence and high costs for health services (21). It is for this reason that research has begun to focus on the concept of “early osteoarthritis” (EOA), with the aim of providing the patient a treatment in the early stages of the disease, which can prevent progression and structural changes in the joint, associated with later stages of OA (22, 23).

It is of prime importance that predictors for knee OA progression are found in order to improve treatment options and, moreover, to prevent this disabling disease (24, 25). However, even though a large number of potential risk factors have been studied, most of the findings should be approached with caution, because of the studies design or controversial results (26). In addition, it is appropriate to analyze whether or not the alteration of different metabolic markers, related to inflammatory processes present in knee OA patients, can help to obtain an early diagnose of the injury, and how their modification can influence its course. Therefore, the aim of this work was to study differences of serum lipid and inflammatory biomarkers with knee EOA in comparison with matched controls, in order to determine the role of these factors in the origin of EOA. The secondary objective is to determine the association of serum lipid and inflammatory biomarkers with pain intensity, disability, and functional variables in patients with knee EOA.

## Methods

### Study design

A cross-sectional study with a non-randomized sample was performed. The design followed the international recommendations for Strengthening the Reporting of Observational Studies in Epidemiology (27). All participants received an explanation of the study procedures, which were planned according to the ethical standards of the Declaration of Helsinki and approved by an Ethics Committee (CEIm La Fe 2017/0147). Written informed consent was obtained from all participants before their inclusion.

### Participants

Subjects were recruited and followed at Hospital La Fe, Valencia, Spain, within the H2020 project OACTIVE. The design of the data collection protocol started in November 2017 and lasted until July 2018.

The subjects recruited were evaluated by a group of three experienced physical medicine and rehabilitation clinicians. The inclusion criteria for EOA patients were based on Luyten’s proposal for EOA classification, as these criteria found a specificity of 76.5% for detection of clinical progression, being valid criteria for research use (28). Criteria were as follows: (a) Patient-based questionnaires: Knee Injury and Osteoarthritis Outcome score (KOOS): 2 out of the 4 KOOS subscales (Pain, Symptoms, Function or Knee-related quality of life) need to score “positive” (≤85%); (b) Patients should present joint line tenderness or crepitus in the clinical examination; (c) X-rays: Kellgren and Lawrence (KL) grade 0–1 standing, weight bearing (at least 2 projections: PA fixed flexion and skyline for patellofemoral OA) (29). For matched controls the inclusion criteria were (a) Patient age greater than or equal to 40 years; (b) Kellgren & Lawrence 0–1.

Exclusion criteria were: (a) Any cognitive disability that hindered viewing of the audio-visual material; (b) Illiteracy; (c) Comprehension or communication difficulties, (d) Insufficient Spanish language comprehension to follow measurement instructions; (e) Presence of any rheumatic, autoimmune or infectious pathology; (f) other current knee injuries diagnosed by magnetic resonance image.

### Outcome measures

#### Serum lipid and inflammatory biomarkers measurement

Serum lipid profile analysis (total cholesterol, triglyceride, HDL, and LDL) and inflammatory biomarkers C-Reactive protein (CRP), and uric acid (UA) were measured in the biochemistry laboratory of the hospital. For the collection of blood we followed the rules proposed by the Standard Operating Procedures Internal Working Group (SOPIWG)/Early Detection Research Network (EDRN) for specimen collection. Four aliquots were sent on dry ice by courier to the Bionos Biotech La Fe and kept at −50°C until measurements of serum were taken using commercial ELISA kits (IDS Co., Bolden, United Kingdom), with intraassay coefficients of variation ranging from 5 to 15%.

### Pain and disability variables

#### Pain intensity

Visual Analogue Scale (VAS) was used to measure pain intensity. The VAS is a 100-mm line with two endpoints representing the extreme states “no pain” (0) and “the maximal pain imaginable” (10). It has been shown to have good retest reliability (*r* = 0.94, *p* < 0.001) and a minimal detectable change of 15.0-mm (30, 31).

#### Western Ontario and McMaster universities osteoarthritis index (WOMAC)

This instrument is the most extensively used for the functional and symptomatic assessment of patients with OA. The WOMAC questionnaire is self-administered and is used to assess patients who progress with hip and/or knee OA. The questionnaire is a multidimensional scale composed of 24 items divided into three aspects: functional pain (consisting of 5 items), stiffness (2 items) and activities of daily life difficulties (17 items). Higher values mean poorer WOMAC subscales scores of pain and physical function. The Spanish version of the WOMAC questionnaire has adequate psychometric properties, presenting an index of internal consistency (a) of 0.82 for pain and 0.93 for physical function subscales (32).

### Functional variables

#### Five times sit-to-stand test

To assess functional capacity, the sit-to-stand test was employed. The test was performed as follows: with the patient seated with their back against the back of the chair, the clinician counted each stand aloud so that the patient remained focused. The clinician stopped the test when the patient achieved the standing position on the 5th repetition (33). The amount of time needed to conduct the test was calculated in seconds. Relative inter-rater reliability results (ICC = 0.937) revealed excellent reliability (34).

#### Gait speed

The subject walked 10 m (32.8 feet) unaided and the time was measured for the intermediate 6 m (19.7 feet). Assistive devices could be used but had to be kept consistent and documented from test to test. It was performed at the fastest speed possible. There were three trials collected and the average of the three trials was calculated for the measurement (35, 36). Gait speed measurements were shown to have excellent test–retest reliability (ICC values of 0.96–0.98) (37).

### Procedures

An information sheet with an explanation of the procedure and an informed consent form were given to all the participants. Once the subject had read the information regarding the study, they were allowed to ask any questions about its nature. Those subjects who agreed to participate proceeded to fill in the sociodemographic questionnaire. Their Self-reported measures of disability, pain and disability self-reported variables were then assessed. The study protocol lasted approximately 1 h. This procedure was identical for both groups.

### Statistical analysis

The sociodemographic and clinical variables of the participants were analyzed. The data were summarized using frequency counts, descriptive statistics, summary tables and figures. The data analysis was performed using the Statistics Package for Social Sciences (SPSS 24, IBM Inc., United States). The categorical variables are shown as frequencies and percentages. The quantitative results are represented by descriptive statistics (confidence interval, mean, and standard deviation). Student’s *t-*test was used for the group comparisons. Cohen’s *d* effect sizes were calculated for multiple comparisons of the outcome variables. According to Cohen’s method, the magnitude of the effect was classified as small (0.20–0.49), medium (0.50–0.79), or large (0.80).

The relationships between serum lipid measures with functional, pain and disability measurements in patients with EOA were examined using Pearson’s correlation coefficients. A Pearson’s correlation coefficient greater than 0.60 indicated a strong correlation, a coefficient between 0.30 and 0.60 indicated a moderate correlation and a coefficient below 0.30 indicated a low or very low correlation (38).

## Results

A total of 96 participants were included in the study, with a mean age of 51.81 ± 5.59 (39 men and 58 women). 48 of the participants met the criteria to be classified as EOA and 48 participants as matched controls for sociodemographic characteristics. No abandonment of any participant was recorded, nor adverse effects were reported during the assessments. Also, there were no statistically significant differences between the groups in terms of descriptive and demographic variables (Table 1).

**Table 1:** Descriptive and demographic variables.

Measures	EOA (*n* = 48)	HS (*n* = 48)	*p* value
Age (years)	52.36 ± 5.02	50.72 ± 6.87	0.46
BMI (kg/m^2^)	26.98 ± 4.36	27.50 ± 3.89	0.43
KL			0.65
0	19 (39.6)	20 (41.6)	
1	29 (60.4)	28 (58.4)	
Gender			0.17
Women	39 (81.3)	43 (89.6)	
Men	9 (18.7)	5 (10.4)	
Economic status			0.86
Easy	8 (16.7)	3 (6.3)	
Fairly easy	35 (72.9)	38 (79.2)	
With some difficulties	3 (6.2)	7 (14.5)	
With great difficulties	2 (4.2)	0 (0)	
Alcohol			0.65
Never	10 (22.2)	8 (16.7)	
Seldom	20 (41.7)	18 (37.5)	
1–2 times/month	4 (8.3)	8 (16.7)	
1–2 times/week	7 (14.3)	12 (25)	
1 time day	5 (10.3)	2 (4.1)	
More than 1 a day	2 (4.1)	0 (0)	
Smoking			0.27
Yes	3 (6.25)	6 (12.5)	
No	10 (22.2)	20 (41.7)	
Ex	35 (72.9)	22 (45.8)	

Data are expressed as mean ± standard deviation or n (%). BMI, Body Mass Index; KL, Kellgren & Lawrence; EOA, Early osteoarthritis.

### Main results

**Figure 1:**
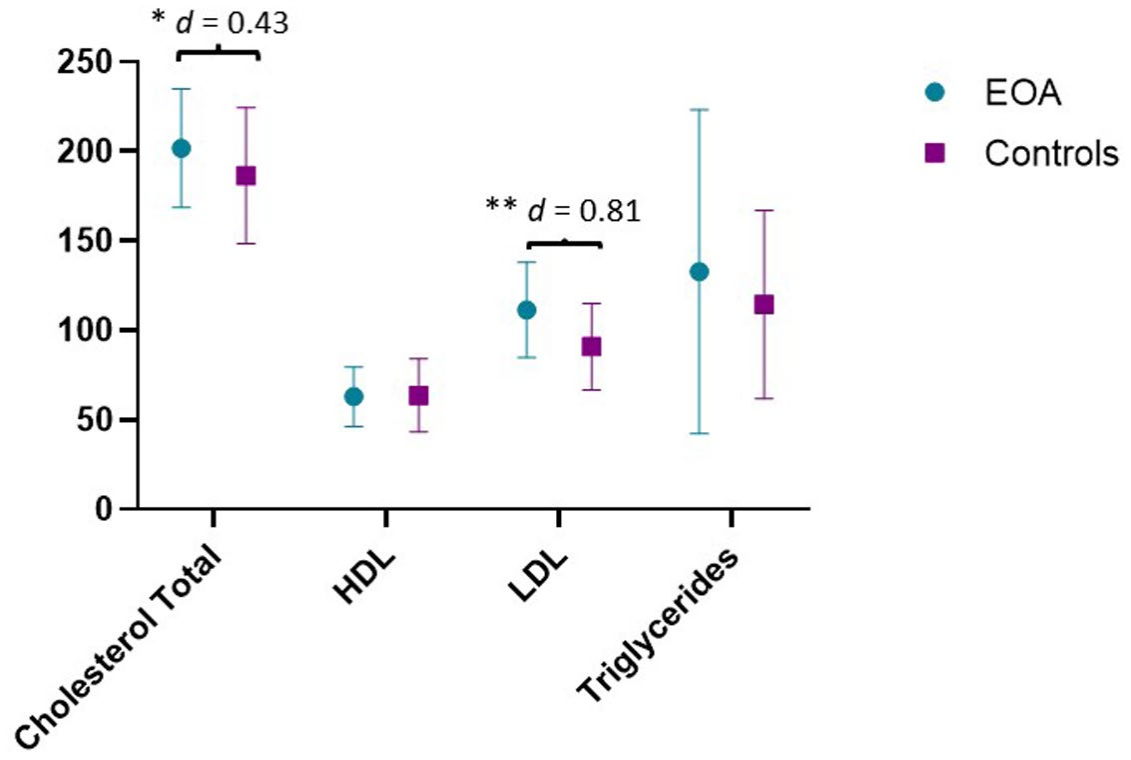
Results of serum lipid profile analysis. Results are expressed in ng/mL. HDL, high-density lipoprotein; LDL, low-density lipoprotein; EOA, Early Osteoarthritis. ^**^p < 0.01; ^*^p < 0.05; *d* = Effect size.

Regarding serum lipid profile analysis, statistically significant differences were found between groups. Patients with EOA showed higher levels of total cholesterol (201.78 ± 33.1 ng/mL) in comparison with matched controls (186.43 ± 38.03 ng/mL). Student’s *t*-test showed statistically significant differences (*p* < 0.05), with medium effect size (MD: −15.36; *d* = 0.43). Similarly, patients with EOA showed higher levels of LDL (11.45 ± 26.59 ng/mL) in comparison with matched controls (90.86 ± 24.0.7 ng/mL). Student’s *t*-test showed statistically significant differences (*p* < 0.05), with large effect size (MD: −20.59; *d* = 0.81). However, no significant differences were found between groups in HDL and triglycerides (Figure 1). Serum lipid profile was compared to reference values according to the Adult Treatment Panel III (ATP III) (39) (Table 2).

**Table 2:** Differences between groups.

Measures	EOA	Controls	Reference values	Mean difference (95% CI)	Effect size (*d*)
Uric acid	5.72 ± 2.6	4.19 ± 1.53	<6.0	−1.53^**^ (−2.37 to −0.7)	0.7135
Cholesterol Total	201.78 ± 33.1	186.43 ± 38.03	<200	−15.36^*^ (−29.49 to −1.22)	0.4313
HDL	62.96 ± 16.51	63.65 ± 20.46	>40	0.69 (−6.80 to 8.17)	0.0370
LDL	111.45 ± 26.59	90.86 ± 24.07	<100	−20.59^**^ (−30.67 to −10.51)	0.8111
Triglycerides	132.71 ± 90.48	114.43 ± 52.63	<150	18.28 (−11.48 to 48.04)	0.2476
C-reactive protein	2.73 ± 2.74	1.78 ± 1.82	<1	−0.95^*^ (−1.88 to −0.02)	0.4098

Data are expressed as mean (mg/dL) ± standard deviation. HDL, high-density lipoprotein; LDL, low-density lipoprotein; EOA, Early Osteoarthritis; CI, Confidence Interval. ^**^p < 0.01; ^*^p < 0.05.

**Figure 2:**
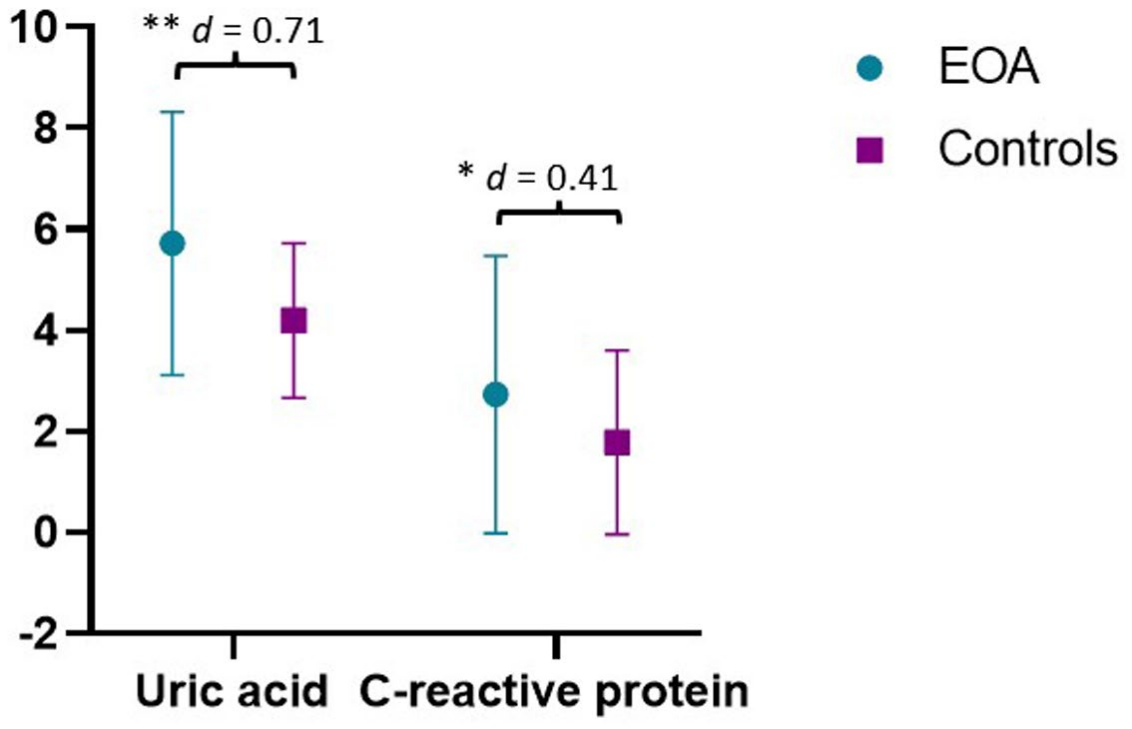
Results of inflammatory biomarkers. Results are expressed in ng/mL. EOA, Early Osteoarthritis. ^**^p < 0.01; ^*^p < 0.05; *d* = Effect size.

In relation to inflammatory biomarkers, patients with EOA present higher levels of UA and CRP, and Student’s *t*-test showed statistically significant differences for both variables (*p* < 0.05), with medium effect size (MD: 1.53; *d* = 0.71 and MD: −0.95, *d* = 0.41, respectively) (Figure 2). Inflammatory biomarkers were compared to reference values previously established (40, 41) (Table 2).

### Correlation analysis

Correlation analysis showed that higher levels of total cholesterol were correlated with higher levels of pain intensity and higher disability, with low to moderate correlation strength (*r* = 0.25 and 0.37; *p* < 0.05). In addition, LDL and CRP levels were correlated with higher pain intensity (*r* = 0.44 and 0.34; *p* < 0.05). Finally, UA and CRP were inversely correlated with gait speed and sit-to-stand tests, with moderate strength (*r* = −0.038 to −0.5, *p* < 0.05) (Table 3).

**Table 3:** Correlation analysis.

	WOMAC	Pain intensity	Walking speed	Sit-to-stand
Uric acid	0.04	0.04	−0.48^*^	−0.39^*^
Cholesterol total	0.25^*^	0.37^*^	0.09	0.02
HDL	0.03	−0.1	0.04	0.08
LDL	0.33^*^	0.44^*^	0.05	0.2
Triglycerides	0.1	0.04	0.2	0.15
C-reactive protein	0.23	0.34^*^	−0.5^*^	−0.38^*^

HDL, high-density lipoprotein; LDL, low-density lipoprotein; EOA, Early Osteoarthritis. ^*^p < 0.05.

## Discussion

The main aim of this work was to study differences of serum lipid and inflammatory biomarkers with knee EOA in comparison with matched controls, in order to determine the role of these factors in the origin of EOA. The main results showed that patients with EOA have higher levels of total cholesterol, LDL, UA, and CRP compared to matched controls.

The relationship between serum lipid levels is contradictory in the current scientific evidence. A systematic review and meta-analysis found that in case–control and cross-sectional studies, there was a clear relationship between dyslipidemia and the presence of OA (case–control: OR = 1:37; 95%; CI = 1:27–1.46; cross-sectional: OR = 1:33; 95%CI = 1:21–1.46), especially in the knee. However, this same review did not find the same relationship when analyzing only cohort studies (RR = 1:00; 95%; CI = 0:85–1.14) (42).

Several mechanisms have been proposed in relation to lipid metabolism and OA. Cholesterol has a crucial role in chondrogenesis and endochondral osteogenesis (43). In this regard, it has been shown that higher levels of HDL may have a protective effect on joints (44). However, inflammatory processes can be triggered by the accumulation of persistent LDLs in the cartilage extracellular matrix. Oxidized LDL ligands to chondrocyte LOX-1 stimulate the production of ROS in chondrocytes, resulting in the expression of catabolic genes (45, 46). Several previous studies show relationships between inflammation and disequilibrium in cholesterol homeostasis, as osteoarthritic cartilage presents remarkable down-regulation of the expression of the cholesterol efflux gene compared to normal cartilage (47, 48). In addition, when there is an aggregation of joint cholesterol, vascular injury is at risk, resulting in impaired blood flow to the subchondral bone. When cartilage loses oxygen and nutrients, this can result in histopathology and contribute to the development of OA (48, 49). Additionally, hypercholesterolemia can also lead to oxidation and lipid deposition in tissues, resulting in cartilage damage (49). Our results showed higher levels of total cholesterol in patients with EOA and a correlation between these levels and a greater intensity of pain and disability, showing a possible relationship between cholesterol and the genesis of OA (metabolic OA phenotype). Furthermore, these results suggest the need to further investigate the relationship between cholesterol levels, inflammation, and clinical symptoms in these patients, so that new therapies take into account pro-inflammatory nutritional aspects that appear to be linked.

CRP is an acute-phase protein synthesized by hepatocytes in response to pro-inflammatory cytokines during inflammatory/infectious processes (50). Low levels of CRP can be measured accurately, and it is thus possible to identify individuals with low-grade inflammation, that is associated with an increased risk of several diseases (51, 52). The involvement of CRP in the pathogenesis of OA in humans has been suggested (53). Systemic CRP levels are significantly elevated in OA patients compared to healthy controls and have been reported to be related to clinical features and radiographic severity (54). In addition, similar to our results, the population-based Chingford study confirmed these findings and the authors suggested that CRP levels in EOA can be used as a predictive marker of disease progression (55). Similarly, the meta-analysis performed by Jin et al. showed that CRP levels were significantly associated with pain and decreased physical function, but not with radiographic OA, similar to our results (56).

In relation to UA, an association between UA and OA has been proposed due to a shared pathogenetic mechanism related to MS. *In vitro* analyses of the effects of monosodium urate crystals on catabolic and proinflammatory signaling in chondrocytes revealed a strong dose-and time-dependent reduction in the viability of primary human chondrocytes and cartilage explants, together with a decrease in cartilage matrix proteins (57). Also, urate crystals significantly increased the expression of catabolic mediators such as nitric oxide (58, 59). In addition to urate crystals, soluble UA has various proinflammatory and prooxidant effects, and the presence of UA transporters in human chondrocytes suggests that they can internalize soluble UA and initiate catabolic responses (60). However, the specific role of uric acid during autophagy is currently unknown, but cell death could result in local supersaturation of uric acid as cytolysis generates large quantities of purines from RNA and DNA (61). As suggested by Matzinger’s theories, the release of uric acid could provide an inflammatory danger signal which is responsible for activating a chronic immune inflammatory response, being the result of activation of IL-18 and IL-1β (62).

Finally, UA and CRP were inversely correlated with gait speed and sit-to-stand tests, these being clinical measures of functionality. Thus, the sit-to-stand test has been directly related to leg strength, a measure of functional status and ability (63). Furthermore, decline in gait speed has been associated with an increased risk of all-cause mortality and other markers of health in well-functioning individuals (64). This finding is interesting given that already in patients with EOA, inflammatory biomarkers have a direct relationship with functionality. In this sense, these functional tests are quick, easy, and low-cost to use in clinical practice, so it may be relevant to assess them for early detection of inflammatory changes and to try to prevent physical decline and disease progression.

### Limitations

Due to the nature of the study (cross-sectional), it is not possible to assess any cause-and-effect relationship neither temporal relationship between inflammatory and serum lipid biomarkers and the clinical variables assessed. In addition, it is not possible to determine the direction of the relationship (e.g., if inflammatory and serum lipid biomarkers levels are the result of the presence of OA or if they are responsible of its incidence). Finally, results should be interpreted with caution due to the low sample size included and the lack of the possibility to calculate the sample size in advance.

## Conclusion

Patients with EOA have higher levels of total cholesterol, LDL, UA, and CRP compared to matched controls. In addition, higher levels of total cholesterol, LDL and CRP were correlated with higher levels of pain intensity and higher disability, and UA and CRP were inversely correlated with functional status. These results highlight the relevance of metabolic and proinflammatory aspects in the early stages of knee OA and could be key to developing early diagnoses to prevent the onset and development of the disease.

## Data availability statement

The raw data supporting the conclusions of this article will be made available by the authors, without undue reservation.

## Ethics statement

The studies involving human participants were reviewed and approved by the La Fé Hospital. The patients/participants provided their written informed consent to participate in this study.

## Author contributions

LH-M, AA-C, EV-H, and IV-A contributed to conception and design of the study. LH-M organized the database. JCl performed the statistical analysis. FC-M wrote the first draft of the manuscript. LS-M, FF-S, JCs, and MB-D wrote sections of the manuscript. All authors contributed to manuscript revision, read, and approved the submitted version.

## Funding

This project has received funding from the European Union’s Horizon 2020 Research and Innovation Program under grant agreement no. 777159.

## Conflict of interest

The authors declare that the research was conducted in the absence of any commercial or financial relationships that could be construed as a potential conflict of interest.

## Publisher’s note

All claims expressed in this article are solely those of the authors and do not necessarily represent those of their affiliated organizations, or those of the publisher, the editors and the reviewers. Any product that may be evaluated in this article, or claim that may be made by its manufacturer, is not guaranteed or endorsed by the publisher.
